# The MicroRNA3686 Inhibits the Proliferation of Pancreas Carcinoma Cell Line by Targeting the Polo-Like Kinase 1

**DOI:** 10.1155/2015/954870

**Published:** 2015-05-21

**Authors:** Hong-Yi Jin, Xin-Guang Qiu, Bo Yang

**Affiliations:** ^1^Department of General Surgery, The First Affiliated Hospital of Zhengzhou University, Zhengzhou University, Zhengzhou 450052, China; ^2^Department of Emergency, Chinese PLA General Hospital, Beijing 100853, China; ^3^Department of Thoracic Surgery, Anyang Tumor Hospital, Anyang 455000, China

## Abstract

The Polo-like kinase 1 (PLK1) is one member of the so-called Polo-like kinase family which plays an important role in tumorigenesis. By analyzing the potential complementary microRNA (miRNA) targeting sequence of PLK1, we identified that miRNA-3686 (hereby and thereafter mir3696) could be the potential regulator for PLK1. Real-time PCR demonstrated that the mir3686 has a relatively higher expression in the immortalized pancreas cell HPDE6C7 than pancreas carcinoma derived cell line PANC1. The upregulation of mir3686 in HPDE6C7 cell corresponded with the low expression of PLK1 as well. Both luciferase based reporter assay and evaluation of endogenous PLK1 expression demonstrated that mir3686 regulated PLK1, which confirms our speculation. Moreover, we found that transfection of mir3686 in PANC1 cell could lead to proliferation inhibition and promote apoptosis. Further analysis demonstrated that mir3686 transfection in PANC1 cell also inhibited cell invasion, and clone formation in cell invasion assay and clonogenic cell survival assay, respectively. In contrast, inhibition of mir3686 expression in HPDE6C7 cell enhanced the capability of proliferation, cell invasion and clone formation. Taken together, our results indicated that mir3686 could target PLK1 to inhibit the cell proliferation in pancreas cancer derived cell line and mir3686 could be a new therapeutic target for pancreas cancer treatment.

## 1. Introduction

The Polo-like kinase 1 (PLK1), also known as the serine/threonine-protein kinase PLK1, or serine/threonine-protein kinase 13 (STPK13), is one member of the Polo-like kinase family [1]. Originally, the* Polo* gene had been identified as a key regulator in mitosis in* Drosophila* [2, 3]. Mutation of* Polo* leads to many defects in mitosis [2, 3]. Lately, the* CDC5* gene of* Saccharomyces cerevisiae* had been confirmed to encode a homologue to* polo *of* Drosophila* which implies that there may be more* Polo* homologues in other species [4]. These earlier studies finally led to the discovery of human* polo* homologue which is named Polo-like kinase 1 (PLK1) [5]. Now we know that* Polo* is a highly conserved gene which is expressed from yeast to human and plays the key role during mitosis, meiosis, and cytokinesis [6]. Several PLKs are present in mammalian species as PLK 1 to 4; however, only one member has been identified in other species, such as* Polo* in* Drosophila* [7].

Analysis had indicated that the PLK1 plays important roles in tumorigenesis since it is functionally related with cell cycle. In normal cell, PLK1 is upregulated from S/G2 phase and reaches the highest activity during mitosis [1, 7]. After that, PLK1 degradation starts in the late stage of mitosis and continues throughout G1 phase [8]. Overexpression of PLK1 in a variety of cancers had been observed [9]. It had also shown that overexpression of PLK1 could lead to the transformation of normal human fibroblasts* in vitro* and xenograft of those PLK1 transformed cells was capable of generating tumors in nude mice [10]. Furthermore, data gained from pancreatic adenocarcinoma patients suggests that dysregulation of PLK1 occurred early in carcinogenesis and overexpression of PLK1 was found in pancreatic intraepithelial neoplasia III lesions [11].

In recent year, the microRNA (miRNA), a small noncoding RNA molecule, had been demonstrated to play an important role in regulating genes expression [12]. Since the target of a single miRNA may be multiple, dysregulation of miRNA expression may profoundly influence cancer-related signaling pathways [13]. For the PLK1 kinase, it had been demonstrated that overexpressed PLK1 in esophageal cancer could be targeted by miRNA-593 [14]. Another group also showed that miRNA-100 could regulate PLK1 in human nasopharyngeal cancer as well [15]. However, the regulation network of PLK1 by miRNA is still largely unknown.

In this study, we identified that the microRNA3686 (mir3686) is a potential regulator for PLK1. Overexpression of mir3686 inhibited the proliferation of pancreas carcinoma derived cell line. In contrast, inhibition of mir3686 in the immortalized pancreas cell line HPDE6C7 could result in the enhanced cell proliferation and clone formation. In sum, our data identified the new microRNA regulator for PLK1 and it could serve as a potential target for therapy.

## 2. Materials and Methods

### 2.1. Cells, miRNA Mimics, and Chemical

HEK293T cell, HPDE6c7 cell, and PANC1 were maintained in Dulbecco's Modified Eagle Medium (DMEM) supplemented with 10% fetal bovine serum (Gibco, Carlsbad, CA, USA). Transfection of HEK293T with plasmid DNA was performed by using Lipofectamine 2000 (Invitrogen, Grand Island, NY, USA), according to the instructions of the manufacturer.

The mirVana miRNA-3686 mimic, inhibitor, and mirVana miRNA-3686 mimic and miRNA Negative Control (scramble control) were all commercially purchased from Life technologies (Carlsbad, CA, USA). Transfection of miRNA mimic was conducted by Lipofectamine 2000 as well.

### 2.2. Western Blot Analysis

Cells were lysed by the Laemmli Sample Buffer as previously described [16, 17]. Cell lysate was analyzed by sodium dodecyl sulfate-polyacrylamide gel electrophoresis (SDS-PAGE) and Western blot as previously described [17]. Briefly, separated proteins in SDS-PAGE were transferred onto PVDF membrane and probed with rabbit anti-PLK1 antibody (Santa Cruz Biotechnology, Santa Cruz, CA, USA). Specific reactions were detected by using goat anti-rabbit IgG conjugated with horseradish peroxidase (Sigma, St. Louis, MO) and revealed by a chemiluminescence substrate. The membrane was also blotted with GAPDH antibody (Santa Cruz) to normalize the protein loading. The chemiluminescence signal was recorded by the ChemiDoc XRS imaging system (Bio-Rad Laboratories, Hercules, CA, USA). Data analysis was conducted by the Quantity One Program (Version 4.6).

### 2.3. Reverse Transcription and Real-Time PCR (qPCR)

Total RNA was isolated from cells with TRIzol Reagent (Invitrogen, Carlsbad, CA, USA). RNase-free DNase (Promega, Madison, Wisconsin, USA) was used to remove DNA from the RNA isolation procedure. Reverse transcription via AMV reverse transcriptase (Promega) was conducted by either a combination of oligo dT and random hexamer or gene specific primer according manufacturer's instruction. Real-time PCR detection with SYBR Green Mix (Life technologies) for the targeting genes was described previously [16, 18]. Transcripts of GAPDH and U6 were also detected from the same samples to serve as an internal control for normalization. Gene expression was quantified by 2^−ΔΔCT^ method [19]. Primers used in this study were listed as Table 1.

### 2.4. Luciferase Reporter Assay

The 3′UTR of PLK1 mRNA containing the mir3686 target sequence was cloned to the XhoI and NotI sites of psiCHECK-2 (Promega) by primers PLK1 3UTR F1 and PLK1 3UTR R1 to generate a PLK reporter. The final PCR product of PLK1 3UTR F1 and R1 is 304 bp. Luciferase activities in the HEV293T cell transfected with PLK1 report plasmids along with mir3686 or scramble miRNA were evaluated by using Dual-Glo Luciferase Assay System (Promega) according manufacturer's instruction. The luminescence signal was measured with a VICTOR3 Multilabel Counter (Perkin-Elmer, Waltham, MA). Relative percentages of luminescence intensity were calculated by comparison with a MOCK-treatment control.

### 2.5. Cell Proliferation Assay (MTT)

The trypsinized cells were stained by trypan blue for counting of viable cells. Then the single cell suspension was seeded in 96-well plates with 2 × 10^4^ cell per well. After overnight incubation, the mir3686 mimic and scramble control were added into each well accordingly. Then the cell proliferation was determined at the indicated time points by using MTS Cell Proliferation Colorimetric Assay kit (Biovision, Milpitas, CA, USA) according manufacturer's instruction.

### 2.6. Flow Cytometry Based Cell Cycle and Cell Apoptosis Assay

A total of 1 × 10^6^ PANC1 cells of each group with mir3686 mimic or scramble control transfection for 48 h were fixed with 70% ethanol and permeabilized by PBS containing 1% TritonX100 (Sigma) and DNase-free RNase A (Sigma). Then the fixed cells were stained by Propidium iodide for cell cycle analysis.

The same amount of cells was treated in the same way as for cell cycle analysis. Then the cells were stained with FITC labeled Annexin V and Propidium iodide. Then the stained cells were analyzed via flow cytometry machine (FACSCalibur, BD Biosciences, San Jose, CA, USA) for cell apoptosis.

### 2.7. Transwell Cell Invasion Assay

To evaluate the capability of cell invasion, the Transwell cell invasion assay had been conducted as previously described with modifications [20]. Briefly, 12 hours before experiment, the cell culture medium had been discarded and replaced with FBS free DMEM for miRNA transfection. 24 hours after transfection, the cell was trypsinized and stained with trypan blue for cell counting. A total of 100 *μ*L cell suspension medium had been added into Transwell chamber and cultured for another 48 h. Then those chambers were stained with hematoxylin. A totally six microscopic fields were randomly selected from each chamber and were captured for quantification.

### 2.8. Clonogenic Cell Survival Assay

Clonogenic cell survival assay was conducted as previously described with modifications [21]. Briefly, miRNA transfected cell was trypsinized and counted. 1 × 10^4^ cells were seeded into 100 mm dishes and maintained for one week. Then the cell colonies were stained with gentian violet for pictures.

### 2.9. Statistical Analysis

The significant differences of mir3686 expression, PLK mRNA expression, luciferase activity, and cell proliferation between the groups of cells in the presence or absence of miRNA mimic were assessed by Student's *t*-tests. A two tailed *P* value of less than 0.01 was considered significant.

## 3. Results

### 3.1. The MicroRNA3686 Was Downregulated in PANC1 Cell and Correlated with PLK1 Upregulation

**Figure 1:**
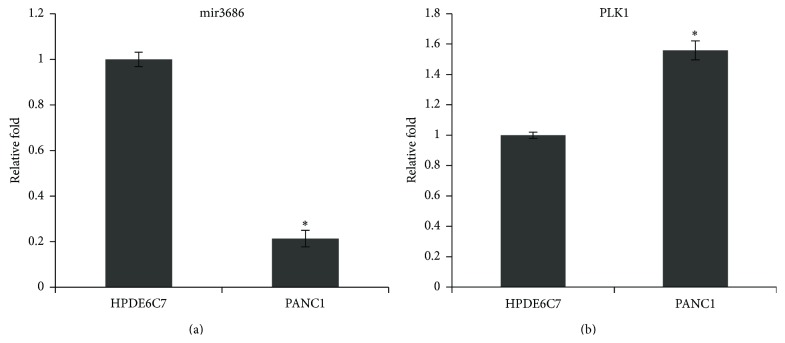
The downregulation of microRNA3686 correlated with upregulation of Polo-like kinase 1 in human pancreas carcinoma cell line. (a) The mir3686 expression in HPDE6C7 cell and PANC1 cell was detected by qPCR. The relative fold was used to compare the expression level. Error bars represent standard errors of three repeated experiments. Significant differences between two cell lines are shown by “*∗*,” which indicates *P* < 0.01. (b) The PLK1 mRNA expression level in HPADE6C7 and PANC1 cell is detected by PCR. The relative fold was used to compare the expression level between two cell lines. “*∗*” indicates significant difference (*P* < 0.01).

The microRNA3686 (hereby and thereafter, mir3686) was originally identified from screening the small RNA library of peripheral blood via deep sequencing [22]. By analysis of the mature sequence of mir3686 which is AUCUGUAAGAGAAAGUAAAUGA (5′-3′), we had identified that the PLK1 could be the potential target as the complementary sequence existed in the 3′UTR of PLK mRNA. Since there were reports that implied the role played by PLK1 in pancreatic cancer and indicated the PLK1 can be targeted by miRNA [11, 14], we first checked the expression level of mir3686 and its relation with the PLK1 expression in a pancreatic carcinoma derived cell line PANC1 cell. An immortalized human pancreatic epithelial cell HPDE6C7 which has the near normal genotype and phenotype was included in here as the control cell line [23]. Our real-time PCR data indicated that the mir3686 expression in PANC1 cell only has 0.2-fold compared with HPDE6C7 cell, suggesting a strong downregulation of mir3686 in pancreatic carcinoma cell line (Figure 1(a)). In contrast, the PLK1 mRNA expression level in PANC1 cell also increased to 1.6-fold which was correlated with downregulation of mir3686 (Figure 1(b)). Taken together, our data suggested that PLK1 mRNA could be the potential target of mir3686 and the increased PLK mRNA level in PANC1 cell may be due to the downregulation of mir3686 expression.

### 3.2. The PLK1 mRNA Is the Target of mir3686

**Figure 2:**
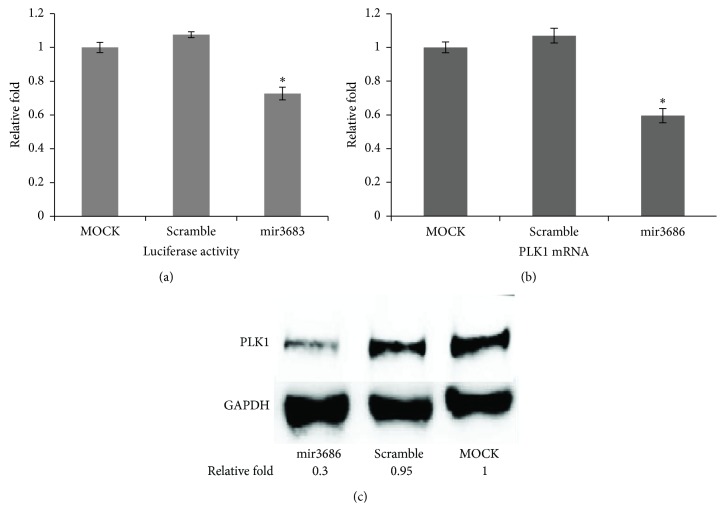
Pole-like kinase 1 (PLK1) is the regulation target of microRNA3686. (a) Luciferase based reporter assay for validating the PLK1 as the target of mir3686. The 3′UTR containing putative mir3686 targeting sequence was cloned to a luciferase report vector and cotransfected to HEK293T cell with mir3686 mimic or scramble control; the luciferase activities was evaluated by Dual-Glo Luciferase Assay System. Error bars represent standard errors of three repeated experiments. Significant differences between groups are shown by “*∗*,” which indicates *P* < 0.01; (b) PLK mRNA expression after mir3686 transfected PANC1 cells. Cells transfected with mir3686 or scramble control for 48 h then the expression of PLK1 mRNA were detected by real-time PCR in different groups. Three repeating experiments had been conducted for each group. Significant differences between groups are shown by “*∗*.” (c) Expression of PLK1 protein in PANC1 transfected with mir3686 and scramble. The Western blotting was done with anti-PLK-1 antibody.

To verify whether PLK1 mRNA is really targeted by mir3686, we conducted reporter assay first. Basically, the 3′UTR region of PLK1 mRNA containing putative targeting sequence of mature mir3686 was cloned to a luciferase vector, and then the reporter vector was cotransfected with mir3686 mimic at the same time to HEK293T cell. 48 hours after transfection, the luciferase activity was analyzed. Compared with the cell transfected with reporter vector alone, cell cotransfected with scramble microRNA control demonstrated minimum effect on luciferase expression (Figure 2(a)). However, with the transfection of mir3686 mimic, there was about 0.2-fold reduction for luciferase activity (Figure 2(a)), suggesting that mir3686 could inhibit the PLK reporter expression. To further confirm our findings, we transfected the mir3686 to PANC1 cell and examined the expression of endogenous PLK1 mRNA as well. Real-time PCR data suggested a 0.4-fold reduction of PLK1 mRNA level compared with normal cell or cell transfected with scramble control (Figure 2(b)). Moreover, Western blot for PLK1 also demonstrated a strong deduction of PLK1 in protein level (Figure 2(c)). In sum, these data confirmed that the mir3686 could target the PLK1 in PANC1 cell.

### 3.3. mir3686 Transfection to PANC1 Cell Leads to Cell Proliferation Inhibition and Promoted Apoptosis

**Figure 3:**
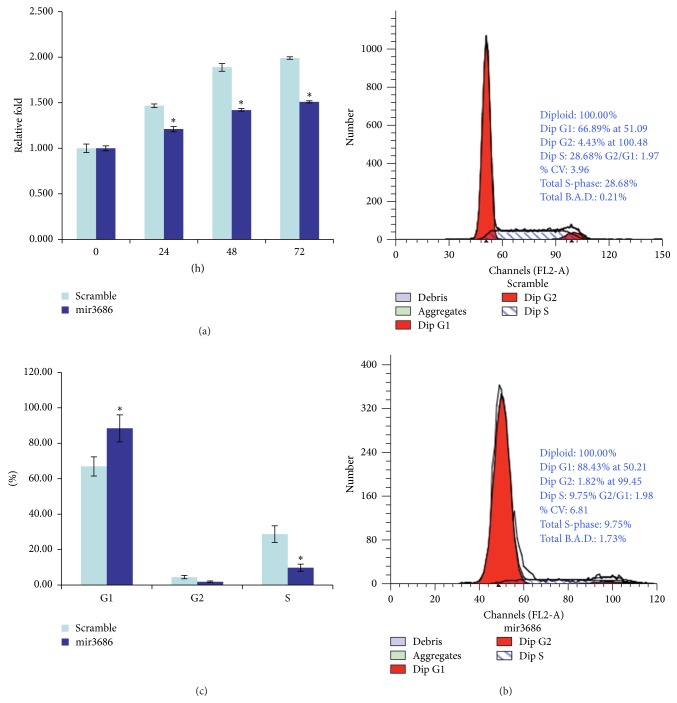
The microRNA3686 is able to inhibit cell proliferation and arrest cell cycle. (a) The cell proliferation assay for mir3686 mimic or scramble control transfected cells. The MTT cell proliferation kit was used to determine the cell proliferation in indicated time points; error bars represent standard errors of three repeated experiments. Significant differences between scramble or mir3686 transfected cells are shown by “*∗*.” (b) Flow cytometry based cell cycle analysis for PANC1 cell transfected with scramble control or mir3686 mimic transfected cell. (c) The quantification of cell cycle from flow cytometry data; three repeating experiments had been conducted for each group. Significant differences between scramble or mir3686 transfected cells are shown by “*∗*” (*P* < 0.01).

**Figure 4:**
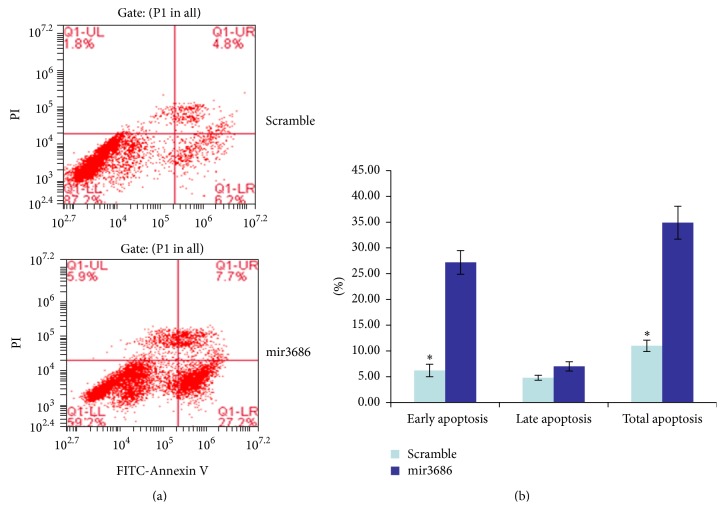
The microRNA3686 is able to promote apoptosis in PANC1 cell. (a) Flow cytometry based apoptosis assay for PANC1 cell. The PANC1 cells were transfected with scramble control or mir3686 and then stained with Annexin V and PI for apoptosis analysis. (b) The quantification of cell apoptosis; three repeated experiments had been conducted for each group. Significant differences between scramble or mir3686 transfected cells are shown by “*∗*” to indicate significant difference (*P* < 0.01).

Since the PLK1 is a key regulator for cell mitosis, we examined whether mir3686 mediated downregulation of PLK1 could inhibit the proliferation of PANC1 cell. Briefly, after the transfection of mir3686 mimic or scramble miRNA, the proliferation of transfected cells was monitored every 24 hours. Compared with the scramble control, which demonstrated the increased proliferation about 1.5-, 1.8-, and 2.0-folds at 24 h, 48 h, and 72 h, the mir3686 mimic transfection resulted only about 1.2-, 1.3-, and 1.3-folds increasing of cell proliferation at corresponding time point, respectively (Figure 3(a)). Furthermore, since it had been reported that PLK1 reaches highest activity during the mitosis, we double checked if inhibition of PLK1 via mir3686 could affect the cell cycle. As demonstrated in Figures 3(b) and 3(c), application of mir3686 arrested more cells (about 88%) in the G1 phase than scramble control group (about 66%). The transfection of mir3686 reduced the cell percentage in G2 and S phase which explained the reduced proliferation of mir3686 transfected cell. Moreover, we also examined the apoptosis status in PANC1 cell transfected with different miRNA mimic. After the miRNA transfection for 48 h, the cells were stained by Annexin V and Propidium iodide for flow cytometry (FCM). FCM analysis for mir3686 transfected cell demonstrated that a total 34.9% of cells was undergoing apoptosis with 27.2% cells within the group were in the early stage pf apoptosis (Figures 4(a) and 4(b)). On the other hand, there was only 11% of the total cells transfected with scramble miRNA that was undergoing apoptosis and 6.2% cells within it were in the early apoptosis (Figures 4(a) and 4(b)). The apoptosis may be another possible explanation for the decreased cell proliferation when PLK1 expression was inhibited by mir3686 mimic transfection.

### 3.4. Transfection of mir3686 in PANC1 Cell Reduced Its Ability to Invasion or Forming New Cell Colony

**Figure 5:**
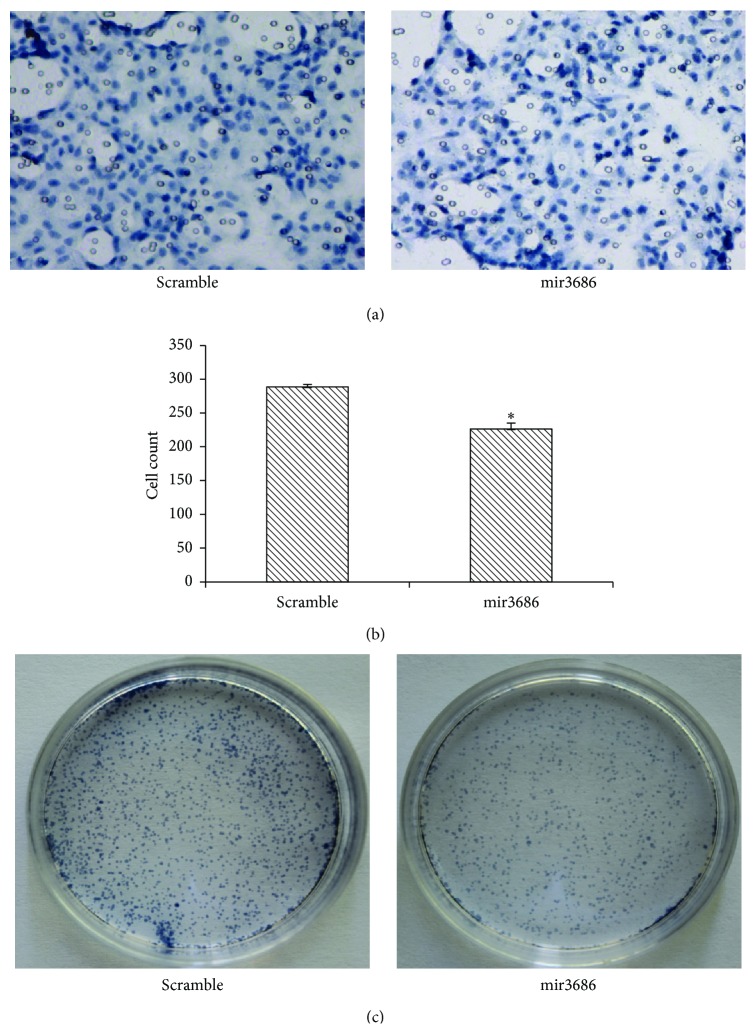
Transfection of microRNA3686 in PANC1 cell reduced its capability of invasion and clone forming. (a) Cell invasion assay picture for scramble control or mir3686 mimic transfected PANC cell. (b) Quantification of cell invasion result between scramble control and mir3686 mimic transfected PANC1 cell indicated the significant different in cell invasion ability. Error bars represent standard errors of six repeated experiments. Significant differences between groups are shown by “*∗*,” which indicates *P* < 0.01; (c) clonogenic cell survival assay indicated mir3686 significantly reduced the colonies formation in mir3686 mimic transfected cell.

Generation of invasiveness of transformed cells such as cancer cell is a necessary step for tumor progression [24]. Since our previous data indicated that cell proliferation could be inhibited via targeting PLK1 by mir3686, we want to know if PLK1 inhibited by mir3686 could reduce the capability of invasion for PANC1 cell. In the cell invasion assay, although cell staining results demonstrated moderate inhibition of invasion for mir3686 transfected cell (Figure 5(a)), by repeating the same assay for six times, we indeed observed the reduction of total cell counting result in the chamber (Figure 5(b)). It looks like that inhibition of PLK1 did not affect the cell invasion ability significantly. However, in Clonogenic Cell Survival Assay which is alternative assay to evaluate the cell proliferation capability, we observed a very strong inhibition of colonies formed by PANC1 cell with mir3686 transfection compared with scramble control (Figure 5(c)). Thus, while PLK1 inhibition resulting a slide deduction of the capability for cell invasion, it still inhibited the colonies formation of PANC1 cell.

### 3.5. Inhibition of MicroRNA3686 Expression in Immortalized Human Pancreas Cell Line Resulted Enhanced Cell Proliferation

**Figure 6:**
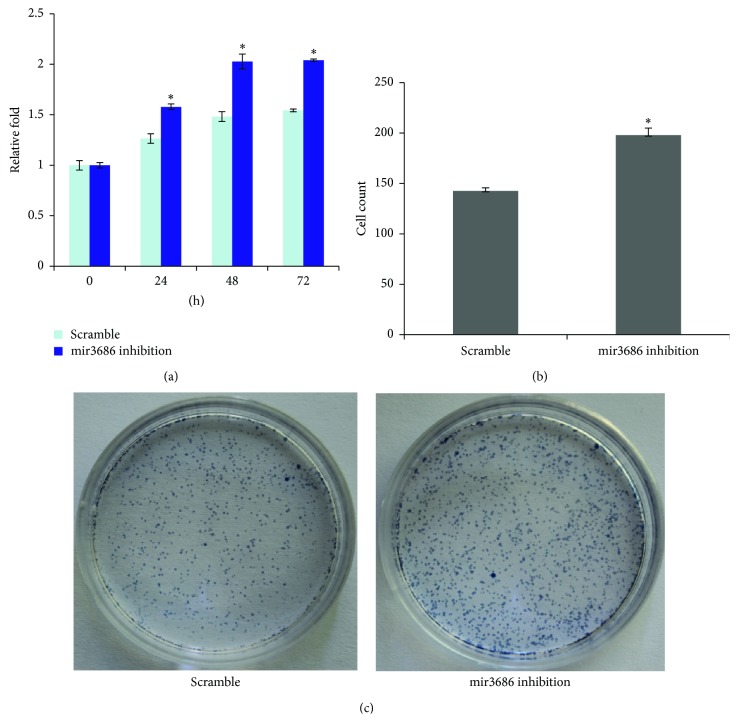
Inhibition of microRNA3686 expression in immortalized human pancreas cell line resulted in enhanced cell proliferation. (a) Cell proliferation assay for mir3686 mimic or scramble control transfected cell indicated time points; each experiment had been conducted for tree times and significant differences between groups are shown by “*∗*,” which indicates *P* < 0.01. (b) The quantification of cell invasion result between scramble control and mir3686 inhibitor transfected HPDE6C7 cell demonstrated that mir3686 inhibition enhance cell invasion. Error bars represent standard errors of three repeated experiments. Significant differences between groups are shown by “*∗*,” which indicates *P* < 0.01; (c) clonogenic cell survival assay indicated that mir3686 increase the cell colonies formation in mir3686 inhibitor transfected HPDE6C7 cell.

As our data had shown that mir3686 expression in the immortalized pancreatic epithelial cell HPDE6C7 is much higher and correlated with decreased expression of PLK1, we wander if inhibition of mir3686 could enhance the proliferation of HAPE6C7 cell. In the MTT based cell proliferation assay, mir3686 inhibition resulted about 1.5-, 2-, and 2-fold increasing of cell proliferation comparing with about 1.2-, 1.5-, and 1.5-fold increasing in control group at the time point 24 h, 48 h, and 72 h, respectively (Figure 6(a)). These data also correlated with the enhanced capability of HPDE6C7 cell to form the colonies in Clonogenic Cell Survival Assay (Figure 6(c)). However, in cell invasion assay, we still only observed the moderate increasing of cell invasion ability (Figure 6(b)). Thus, in those “gain of function” assays, we did enhance the cell proliferation via inhibiting mir3686.

## 4. Discussion

Since the first discovery of* Polo* gene in* Drosophila *andthe key roles it played in mitosis, a lot of studies had been conducted to reveal the function of* Polo* homologues in human [7]. As a lot of evidence suggested the human* Pole* homologue PLK1 plays important role in tumorigenesis, there was still debate to whether overexpression PLK1 in some cancer is the consequence or the cause of cancer [9]. However, there was report that demonstrated PLK1 expression could be target by miRNA to regulate the cell proliferation in esophageal cancer, which implied that PLK1 could be used as the therapeutic target [14].

In this study, we demonstrated that PLK expression was different in immortalized pancreas cell line HPDE6C7 and pancreas carcinoma derived cell line PANC1. Those decreased PLK1 expression may be due to the evaluated expression of microRNA3686, a putative regulator for PLK1. To confirm these findings, both* in vitro *and* in vivo* analysis had been conducted to verify whether PLK1 could be the genuine target for mir3686. Although the PLK1 mRNA expression in mir3686 mimic transfected cell was moderately decreased, the difference between two groups was significant and correlated with the decreased PLK1 in protein level. As microRNA could regulate multiple targets [25], it is possible that the mir3686 mimic we used in this study could target other genes as well; thus it will result in less specificity and moderate inhibition for PLK1. On the other hand, since we only transfected the cell with mir3686 mimic for one time, the stronger inhibition for PLK1 by mir3686 could be achieved by increasing the dose we used for transfection or transfecting the cells for multiple times.

As many studies had documented the important roles of PLK1 in cell proliferation in both normal cell and transformed cell, it had been demonstrated that the inhibition of PLK1 in glioblastoma could result in decreased proliferation by arresting cell cycle and leading to cell death [26]. In our study, by inhibiting the PLK1 expression via mir3686 mimic, we observed the reduced proliferation of pancreas cancer as well. In particular, in addition to inhibition of cell proliferation, pancreas cancer cell transfected with mir3686 demonstrated enhanced apoptosis. Several reports had documented that inhibition of PLK1 in variety cancer cell lines could result in growth reduction and induce apoptosis [27, 28]; however, the detailed mechanism of how PLK1 interaction with the cellular apoptosis pathway is still known and needs further investigation. In addition to a previous report that demonstrated that overexpression of PLK1 had been observed in the early stage of pancreas cancer, our data suggested that PLK1 might be a promising target for the treatment of pancreas cancer.

To further confirm the mir3686 as the potential inhibitor for PLK1 and PLK1 depending on cell proliferation, we conduced cell invasion assay and colony forming assay to evaluate the putative function of mir3686. However, in contrast with the stronger inhibition for the new colonies formed by mir3686 transfected cancer cells, application of mir3686 only moderately reduced the cell invasion ability of cancer cell. Since the 90% of the malignant human tumors are transformed epithelial cells, the invasion ability could enable the malignant cell to break through the basement membrane and invade the underlying mesenchyme [24]. However, research suggested that loss of uvomorulin-mediated cell adhesion plays the indispensable role for the cell to invade its peripheral tissues [24]. Thus, it suggested that the cell invasion is independent of the cell cycle; this may give us a possible explanation why inhibition of PLK1 only partial reduced the invasion capability of cancer cell.

Since we conduced most of our experiment to test the regulation of mir3686 to PLK1 in pancreas cancer cell line PANC1 and the immortalized pancreas cell HPDE6C7 cell demonstrated a stronger expression of mir3686, we performed the “gain of function” assay in HPDE6C7 cell to confirm our previous finding in another way. Our data suggested that inhibiting the mir3686 via its inhibitor did enhance the cell proliferation and colony formation, which is consistent with previous report about the role PLK1 played in pancreas cancer. In conclusion, our study demonstrated that mir3686 is the new regulator of the PLK1 and PLK1 inhibition via mir3686 and could be used as a new target for pancreas cancer therapy.

## Figures and Tables

**Table 1:** Primer.

Primer name	Sequence
miR-3686 RT	CTCAACTGGTGTCGTGGAGTCGGCAATTCAGTTGAGTCATTTACTT
miR-3686 F	ACACTCCAGCTGGGATCTGTAAGAGAA
miR-3686 R	TGGTGTCGTGGAGTCG
U6 F	CTCGCTTCGGCAGCACA
U6 R	AACGCTTCACGAATTTGCGT
PLK1 F	TCCAGGATCACACCAAGCTCAT
PLK1 R	TCGTCGATGTAGGTCACGGCT
GAPDH F	CAGCCTCAAGATCATCAGCA
GAPDH R	TGTGGTCATGAGTCCTTCCA
PLK1 3UTR F1	CCGctcgagTAGCTGCCCTCCCCTCCG(XhoI)
PLK1 3UTR R1	ATAAGAATgcggccgcGAATATTCACATCTGTTTAATGTGCA(NotI)
